# Scalable Production of Monodisperse Functional Microspheres by Multilayer Parallelization of High Aspect Ratio Microfluidic Channels

**DOI:** 10.3390/mi10090592

**Published:** 2019-09-10

**Authors:** Casper Ho Yin Chung, Binbin Cui, Ruyuan Song, Xin Liu, Xiaonan Xu, Shuhuai Yao

**Affiliations:** 1 Department of Mechanical and Aerospace Engineering, The Hong Kong University of Science and Technology, Hong Kong 999077, China; 2 ThunderBio, Hong Kong 999077, China; 3 Bioengineering Graduate Program, Department of Chemical and Biological Engineering, The Hong Kong University of Science and Technology, Hong Kong 999077, China

**Keywords:** droplet spontaneous generation, multilayer device, microsphere synthesis, monodispersity, scalable production

## Abstract

Droplet microfluidics enables the generation of highly uniform emulsions with excellent stability, precise control over droplet volume, and morphology, which offer superior platforms over conventional technologies for material synthesis and biological assays. However, it remains a challenge to scale up the production of the microfluidic devices due to their complicated geometry and long-term reliability. In this study, we present a high-throughput droplet generator by parallelization of high aspect ratio rectangular structures, which enables facile and scalable generation of uniform droplets without the need to precisely control external flow conditions. A multilayer device is formed by stacking layer-by-layer of the polydimethylsiloxane (PDMS) replica patterned with parallelized generators. By feeding the sample fluid into the device immersed in the carrying fluid, we used the multilayer device with 1200 parallelized generators to generate monodisperse droplets (~45 μm in diameter with a coefficient of variation <3%) at a frequency of 25 kHz. We demonstrate this approach is versatile for a wide range of materials by synthesis of polyacrylamide hydrogel and Poly (l-lactide-co-glycolide) (PLGA) through water-in-oil (W/O) and oil-in-water (O/W) emulsion templates, respectively. The combined scalability and robustness of such droplet emulsion technology is promising for production of monodisperse functional materials for large-scale applications.

## 1. Introduction

Microparticles and microspheres made of polymers, hydrogels, and functional materials are useful for a wide range of applications including cosmetics [1], oil and food processing [2], diagnostics [3], pharmaceutics [4], electro/optical agents [5], and catalysts [6]. Conventional manufacturing processes, such as precipitation [7], emulsion polymerization [8], and membrane extrusion [9], have a high production rate and volume but lack control over size and monodispersity. In contrast, droplet-based microfluidics provides precise control of multiphase flow dynamics in confined micro-channels and thus enables the formation of micro-droplets with controlled size, composition, and morphology [10]. Various single-emulsion (e.g., water-in-oil or oil-in-water) and complex-emulsion systems [11] have been developed, offering high throughput microreactors for biological assays [12,13] or uniform templates for synthesis of microparticles [14]. However, the low production rate of microfluidic devices remains a hurdle to bringing the promising yet laboratory-scale functional materials synthesized by microfluidics to the commercial-scale for industrial applications [15].

Many attempts have been made to operate the microfluidic droplet generators in parallel to enhance the throughput of production [16,17]. In flow-focusing [18] or T-junction [19] type geometries, as the droplet break-up is induced by the viscous shearing force of the two phase flows, the pressure fluctuations may cause instability of the shear force and result in polydispersed droplets [20]. Therefore, parallelization of such droplet generators requires relatively complicated configuration to decouple the influence of the connection channels for delivering the two phase flows. For instance, Nisisako et al. [21] arranged a planar square chip composed of parallel droplet generators in a circular configuration, and a cubic supporting module with coaxial annular channels for supplying fluids evenly to the inlets of the mounted chip. Conchouso et al. [22] manufactured parallelization devices using high-precision computer numerical control micro-milling for accurate pattering of the layouts of cap layer, oil-distribution network, water-distribution network, and droplet generation layer. More recently, Yadavali et al. [23] used microfabrication technology to fabricate an all silicon and glass device for very large-scale droplet integration. The throughput can be much enhanced, but the fabrication process involved four mask layers for droplet makers, underpass channels, vias, and delivery channels. Thus, these devices require sophisticated design and fabrication process, and precise alignment for integration. Another type of droplet break-up takes place in structures by variations of channel confinement, such as step emulsification [24,25], gradient of confinement [26], and edge-based droplet generation [27] devices, through which a Laplace pressure difference occurs to induce the formation of uniform droplets. In these structures, droplet pinch-off is predominately driven by the interfacial tension between two phases instead of the shear forces, therefore, the interference of flow or pressure fluctuations can be avoided [28]. On this account, it is easy to parallelize step emulsifiers to enhance the throughput [29,30]. Nevertheless, the fabrication of such devices (e.g., gradient of confinement [26], edge-based or height difference step emulsion devices [25,27]) is still relatively complicated because it needs to achieve different depths in the same device, thus requiring multi-step photolithography.

Our strategy is the parallelization of the droplet generators in a multilayer device for upscaling the production. The basic unit of the droplet generator is a high aspect ratio rectangular nozzle structure, which was previously reported by us to achieve droplet self-emulsification by the confinement and abrupt change of the high aspect ratio geometry of the nozzle and opening to the connection chamber [31]. As the droplet generation is driven by the interfacial tension in this droplet generator, the flow interference is decoupled and the resulting droplet size is solely determined by the width of the nozzle and is not sensitive to flow fluctuations, which enables facile and massive parallelization of such basic units in a single-layer device. In this study, we demonstrate the scalability of such droplet generators by simply stacking three single-layer devices into a multilayer device to further enhance the droplet generation throughput. The multilayer device is facile for operation by applying the sample fluid into the sole inlet, which could be adopted in industrial usage. We also show that, under a critical capillary number, the device can stably generate monodisperse droplets without being affected by the flow interference or fluid properties. Therefore, our device is applicable for both aqueous-based and oil-based emulsions. To demonstrate the application of the device for synthesis of functional microspheres, we used this device to generate two types of materials, the aqueous-based polyacrylamide hydrogel and the organic solvent dissolved Poly (l-lactide-co-glycolide) (PLGA), for microsphere synthesis through water-in-oil (W/O) and oil-in-water (O/W) emulsion templates, respectively.

## 2. Materials and Methods 

### 2.1. Device Fabrication and Operation

The microfluidic device was made of polydimethylsiloxane (PDMS) (Sylgard 184, Dow Corning Corporation, Midland, MI, USA) by a micromolding process [32,33]. The silicon wafer was coated with SU8 photoresist (Model 3050 MicroChem, Westborough MA, USA) in a thickness of 50–85 μm, and the device layout was patterned by photolithography (SUSS Microtec MA6, Garching, Germany). After development (SU-8 developer; MicroChem, Westborough, MA, USA), the silicon wafer with SU8 photoresist yielded a mold for PDMS replica molding. The silicon wafer master mold was put in a vacuum desiccator with 80 μL of Trichloro (1H,1H,2H,2H-perfluorooctyl) silane 97% (Sigma-Aldrich, St. Louis, MO, USA) to modify the surface to become hydrophobic, making it easy to release the PDMS from the mold. A PDMS mixture was prepared by mixing the monomer and curing agent (Sylgard 184, Dow Corning Corporation, Midland, MI, USA) at a 10:1 w/w ratio. After being degassed in a vacuum chamber, the PDMS mixture was poured onto the mold and cured on a hot plate at 80 °C for two hours. The cured PDMS layer was then peeled off from the mold and cut into individual devices. For making a multilayer device, we exposed the bonding surfaces of the PDMS replica to oxygen plasma for 1.5 min and then aligned and assembled the PDMS replica under a microscope. After that, the assembled pieces were baked at 80 °C for 5 h. The inlet ports were punched through all the PDMS layers using a 1.2 mm biopsy punch (Harris Uni Core, Qiagen, Hong Kong, China) connected with tubing (1/8″ OD (outer diameter), 1/16″ ID (inner diameter)) for applying the sample solution. Finally the patterned side of a single layer or a multilayer device and a glass slide (25 mm × 25 mm, Sail Brand, Shanghai, China) were treated with oxygen plasma for 1.5 min and then bonded together. The bonded device was baked at 80 °C for 5 h. For generating water-in-oil droplets, the devices were baked on a hot plate to recover the surface hydrophobicity. Milli-Q water (18 MΩ/cm, Millipore CO., Burlington, MA, USA) was used as the dispersed phase and HFE7500 (3M Novec 7500 Engineered Fluid, Flurochem, Derbyshire, UK) with 1.5% (w/w) fluorosurfactants (ThunderBio, Hong Kong, China) as the continuous phase. To adjust the viscosity of the disperse phase, glycerol (Acros Organics, Pittsburgh, PA, USA) was mixed with Milli-Q water from 0% to 50% (v/v). For generating oil-in-water droplets, the devices were immersed in Milli-Q water after bonding to keep all channel surfaces hydrophilic [34,35,36]. The dispersed phase was HFE7500 and the continuous phase was 2% (wt/v) polyvinyl alcohol (PVA) (Sigma-Aldrich, St. Louis, MO, USA) in Milli-Q water. The single layer or multilayer device was put in the plastic box, followed by the continuous phase to immerse the device, which was held stationary with no external flow. Lastly the dispersed phase was injected into the device at the inlet using syringe pumps, PHD 2000 (Harvard Apparatus, Holliston, MA, USA). This setup was used for all experiments unless otherwise noted. The droplet size and size distribution of the droplets and particles were measured and analyzed using microscope images and a homemade MATLAB program.

### 2.2. Materials

Acrylamide solution (AA; 40% (wt/wt)), AA/bis-acrylamide solution, 40% (wt/wt), molar ratio 19:1 (AA/BIS), N,N,N′,N′-Tetramethylethylenediamine (TEMED), ammonium persulfate (APS), Span-80, polyvinyl alcohol (PVA) (MW 30 000–70 000, 89% hydrolyzed), Poly (L-lactide-co-glycolide) (PLGA) (RG502H, MW 7000–17 000), mineral oil (BioReagent for molecular biology), and chloroform were purchased from Sigma-Aldrich (St. Louis, MO, USA). Dimethyl carbonate (DMC), glycerol, and hexane were obtained from Acros Organics (Pittsburgh, PA, USA). The 1H,1H,2H,2H-Perfluorooctanol was purchased from Alfa Aesar, Shanghai, China. 3M Novec 7500 Engineered Fluid was purchased from Flurochem, Derbyshire, UK. Fluorosurfactants were obtained from ThunderBio, Hong Kong, China. Lipids of 1,2-distearoyl-sn-glycero-3-phosphocholine (DSPC), 1,2-dipalmitoyl-sn-glycero-3-phospho-(1′-rac-glycerol) (sodium salt) (DPPG), and 1,2-distearoyl-sn-glycero-3-phosphoethanolamine-N-[methoxy(poly(ethylene glycol))- 2000] (DSPE-PEG) were purchased from Avanti Polar Lipids (Alabaster, AL, USA). All aqueous solutions were prepared in Milli-Q deionized water (18 MΩ/cm, Millipore CO., Burlington, MA, USA).

### 2.3. Hydrogel Microsphere Preparation

The polyacrylamide hydrogel beads were prepared using a W/O emulsion, polymerization, and were washed [37,38]. The dispersed phase was composed of 6.2% (v/v) acrylamide, 0.18% (v/v) bisacrylamide, 0.3% (wt/v) ammonium persulfate. The continuous phase was HFE7500 oil with 0.4% (v/v) TEMED and 5% (w/w) fluorosurfactant. After droplets were generated in the microfluidic device, the droplets were collected in a container prefilled with 1 mL mineral oil to prevent evaporation, then incubated at 65 °C on a hotplate for 12 h to allow the polymerization of beads. The resulting solidified beads were washed twice with 1 mL of 20% (v/v) 1H,1H,2H,2H-perfluorooctanol in HFE7500 oil and twice with 1 mL of 1% (v/v) Span 80 in hexane and finally centrifuged at 4000 g for 1.5 min. After centrifugation the hexane phase was aspirated. To remove traces of hexane, the beads were washed three times with 1 mL Milli-Q water at 5000 g for 30 s and then re-suspended in 1 mL Milli-Q water. 

### 2.4. Polymer Microsphere Preparation

Poly (L-lactide-co-glycolide) (PLGA) microspheres were produced by formation of droplets followed by solvent evaporation. PLGA was dissolved in organic solvent dimethyl carbonate (DMC). DMC is immiscible with water that dissolves PLGA and is biodegradable and less toxic than other conventional organic solvents. The disperse phase consisted of 50 mg/mL PLGA in DMC, and the continuous phase was formed of Milli-Q water with 2% (v/v) lipid. The lipid solution was used as a surfactant stabilizing droplets to prevent coalescence. It was synthesized according to the following steps. Briefly, 10 mg of 1,2-distearoyl-sn-glycero-3-phosphocholine (DSPC), 5 mg of 1,2-dipalmitoyl-sn-glycero-3-phospho-(1’F-rac-glycerol) (sodium salt) (DPPG), and 5 mg of 1,2-distearoyl-sn-glycero-3-phosphoethanolamine-N-[methoxy(poly(ethylene glycol))- 2000] were dissolved in 5 mL of chloroform in a glass vial. A white thin film was obtained on the glass vial by removing the solvent using a nitrogen stream. A 5 mL portion of 10% (wt/v) glycerol solution was added into the glass vial to hydrate the lipid film followed by incubation at 65 °C for 30 min. The formed lipid solution was allowed to cool to room temperature. Monodisperse polymer droplets were generated by emulsification of the PLGA solvent solution in water, where the lipid served as a stabilizer at the interface between the droplet and water. PLGA particles were obtained after DMC rapidly evaporated.

## 3. Results and Discussion

### 3.1. Working Principle and Device Design

**Figure 1:**
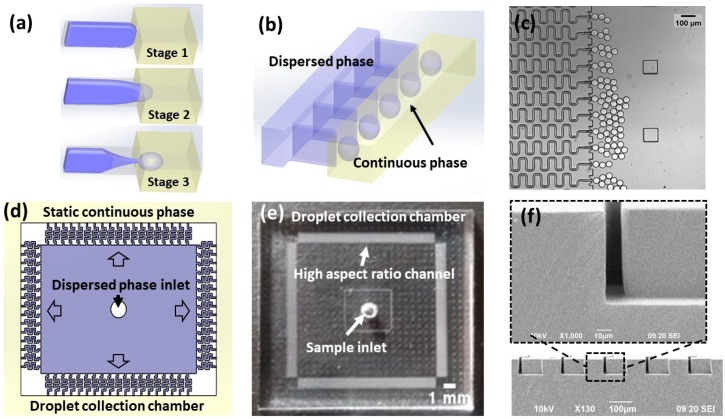
Schematic diagram and photographs of the high-aspect-ratio-induced self-breakup (HIDS) device: (**a**) Schematic showing the droplet breakup process by Plateau–Rayleigh instability in a single HIDS structure where the dispersed phase (purple) is confined in an energy-unfavorable shape. When the channel opens wide at the end, the confined shape is released and the interfacial tension drives the dispersed fluid through elongation, thread thinning, and breakup into droplets in the continuous phase (yellow). (**b**) Schematic showing the parallel integration of an array of the HIDS generators. (**c**) A micrograph of parallelized HIDS generators and generated monodispersed droplets. (**d**) A layout of a parallelized generator device indicating the sample applied at the center and emulsified into the continuous phase through the HIDS generators and collected in the droplet collection chamber in peripheral. (**e**) A photograph of the device with a simple sample inlet and HIDS generators indicated by arrows. (**f**) A SEM image showing the cross-section of the channels with a width of 13 μm and height of 65 μm.

In our previous work [31], we have shown droplet self break-up in a high aspect ratio rectangular microchannel connecting to an open chamber of the same height. The mechanism of the high-aspect-ratio-induced droplet self break-up (HIDS) is illustrated in Figure 1a. Initially, due to the narrow channel confinement, the disperse phase fluid is forced to follow the high aspect ratio geometry in an energy-unfavorable shape (stage 1). When the thread arrives at the nozzle with an opening junction, the strong confinement is released in the horizontal direction. Due to the abrupt change, the equilibrium status of the Laplace pressure cannot be maintained, and the thread elongates and starts necking (stage 2). Triggered by Plateau–Rayleigh-type instability, droplets are pinched off from the thread (stage 3). The fluid interface is restored to the initial shape of the curvature. As the interfacial tension is the dominant force in the HIDS process, the droplet formation is not interfered with by the viscous shearing of the two phase flows (i.e., insensitive to the flow or pressure fluctuations). Moreover, such a droplet generator has a very simple geometry and is easy to fabricate. We can parallelize the HIDS structures in a compact configuration (Figure 1b) for high throughput droplet production. Figure 1c is a micrograph of the droplet generation from an array of such HIDS structures. We further designed a square-shaped device composed of 400 HIDS generators on each side of the device as shown in Figure 1d. With a gentle pressure source, the sample applied at the center inlet spreads outward into the HIDS structures. Through the HIDS structures, the dispersed phase is emulsified into monodispersed droplets in the peripheral chamber, prior to which was filled with the continuous phase. As we have shown that the continuous phase flow rate does not affect the droplet generation [31], the continuous phase can even be static. To collect the generated droplets, we can leverage the flow of the continuous phase by external force or the buoyancy force due to the density difference of the dispersed and continuous phases. For example, using the buoyant force, the droplets may float at the top after being generated. The droplets are then guided to float away to prevent aggregation at the channel outlets. Figure 1e shows a photograph of the device made of PDMS, and Figure 1f is a scanning electron microscope (SEM) photograph showing the cross-sectional view of a rectangular channel with a high aspect ratio of 5 (a height of ~65 μm and a width of ~13 μm).

### 3.2. Characterization of Water-In-Oil and Oil-In-Water Droplets

**Figure 2:**
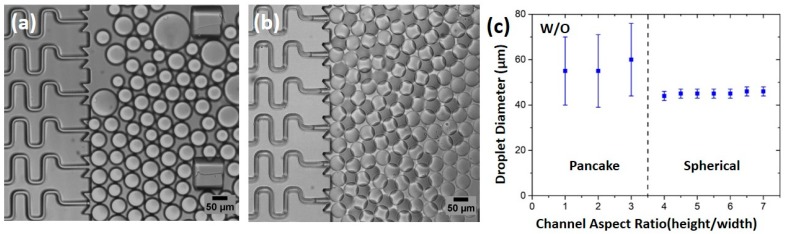
The experimental results of the water-in-oil (W/O) droplets generated from channels with different aspect ratios. (**a**) A micrograph showing polydisperse droplets generated from channels with an aspect ratio of 2. Some big droplets are pancake shaped. (**b**) A micrograph showing uniform droplets generated from channels with an aspect ratio of 4. (**c**) Droplet diameter versus the channel aspect ratio (height/ width). Droplets have a spherical shape and monodisperse with coefficient of variation (CV) <3% for channel aspect ratios 4 and above. The droplet diameter is about 3.5 times the channel width and is not affected by the channel depth. The flow rate of the dispered phase is 1 mL/h and the oil phase is static.

We conducted a series of W/O and O/W emulsification experiments to examine the HIDS droplet generation mechanism and operation condition. To assess the self-emulsification process and the droplet uniformity, we first varied the channel aspect ratio (height/width) of the HIDS generators, in which the width of the nozzle was fixed at 13 μm and the height was varied from 13 μm to 91 μm (the corresponding aspect ratio from 1 to 7). Milli-Q water was used as the dispersed phase and HFE7500 with 1.5% (w/w) fluorosurfactants as the continuous phase. To ensure the droplet’s spontaneous breakup in the interfacial evolution mode, both phases need to be kept at low flow rates. The flow rate applied at the dispersed phase was 1 mL/h (2.5 μL/h for each channel), and the continuous phase remained static. Polydispersed droplets were generated from the channels of aspect ratios ~1 to 3 (Figure 2a), while monodispersed droplets were generated continuously for aspect ratios of 4 to 7 (Figure 2b). The droplet size variation with the channel aspect ratio is shown in Figure 2c. For channel aspect ratio greater than 4, the droplets were measured as ~45 ± 2 µm in diameter with a coefficient of variation (CV) 2.6%. We found the resulting droplet diameter was approximately 3.5 times of the channel width and invariant with the height of the channel, This scaling law has been reported by Xu et al. [31] for W/O emulsion by varying the channel width, which ensures that the droplets remained in a spherical shape for the devices with channel aspect ratios >3.5 [31]. However, when the channel aspect ratio was below 3.5, the break-up of the droplets became irregular and difficult. Sometimes the dispersed fluid thread grew even bigger and formed a pancake shape in a shallow channel before it snapped off, resulting in highly polydispersed droplet population. Therefore, in the following experiments, we used a device with the aspect ratio of 5 to safeguard spherical droplets that were generated as microsphere templates.

Typically, the Capillary number (Ca) is used to classify the droplet generation modes [39,40,41]. Ca represents the relative importance of the viscous force to interfacial tension force acting across an interface between two immiscible fluids. The relation between the droplet break-up modes and Ca, and the critical Ca for transitions between different modes have been investigated in cross-flow [18,42], co-flow [43], and flow-focusing [44] geometries, as well as in step emulsification [45,46]. In this work, we used the capillary number of the dispersed phase, defined as Ca = μV/γ, where μ is the dynamic viscosity of the glycerol fraction in water, V is the velocity of the dispersed phase and γ is the interfacial tension of the two phases.

**Figure 3:**
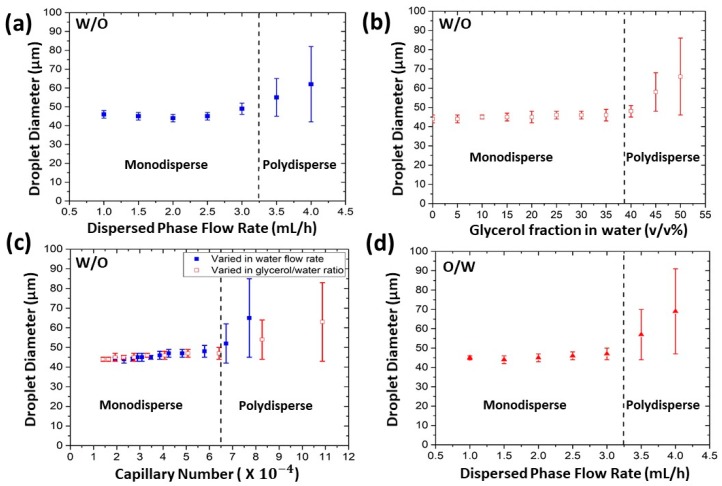
The experimental results of the W/O and oil-in-water (O/W) droplets in various conditions. (**a**) W/O droplets diameter versus the flow rates of the dispersed phase. The droplet size is insensitive for varying dispersed phase flow rates when it is below the critical value (indicated by the dashed line in the figure). (**b**) W/O droplet diameter versus the fraction of the glycerol in water mixture ratio. The flow rate of the dispersed phases is 1.5 mL/h. (**c**) W/O droplet diameter versus the capillary number. The capillary number was calculated based on the dispersed phase flow rate. The data from varied water flow rate (blue) and varied glycerol in water ratio (red) agreed well, indicating a critical Ca of 6.5 × 10^−4^ for uniform droplet generation. (**d**) O/W droplet diameter versus the flow rate of the dispersed oil phase.

To examine how the Ca influences the operational conditions of the HIDS mode, we varied the dispersed phase flow rate from 1 mL/h to 4 mL/h. The result is shown in Figure 3a, where the droplet size became monodisperse at the range from 1 mL/h to 3 mL/h. It shown the droplet size is insensitive to flow rate change but, above the critical flow rate ~3.2 mL/h, the droplet size became polydisperse because some of the generators produced larger droplets resulting in polydisperse droplets. 

In another experiment, we changed the dispersed phase composition by changing the volume ratio of glycerol. The viscosity of the glycerol/water mixture varies from 1.005 to 6.856 for glycerol/water fraction varying from 0% to 50% (v/v) (Table 1) [47,48]. As shown in Figure 3b, the droplet size remained unchanged when the fraction of glycerol was varied within 0% to 42%. Beyond 42%, the mixture was too viscous to operate. It easily clogged the channels, so the severe flow fluctuation made the droplets become polydisperse. We calculated the Ca based on the dispersed phase viscosity and speed in the channel and the interfacial tension of the two phases. The interfacial tension of the water and HFE7500 with fluorosurfactant 1.5% (w/w) was measured as 3.9 × 10^−3^ N/m by Biolin Theta Contact Angle Meter (Biolin Scientific, Theta Lite, Manchester, UK). For the glycerol/water mixtures as the dispersed phase, the variation in the interfacial tension is negligible [49]. We found the critical capillary number for monodisperse droplet generation was about 6.5 × 10^−4^ (Figure 3c), by comparing the data from Figure 3a,b, suggesting the conditions for HIDS shall be controlled below the critical Ca. 

We also repeated these experiments for O/W emulsion where the dispersed phase was HFE7500 and the continuous phase was 2% (wt/v) PVA in Milli-Q water. The observations were consistent with those obtained in the W/O experiments. Figure 3d shows the droplet diameter versus the flow rate of the dispersed phase and ensures the HIDS mode for O/W emulsions was similar to that for the W/O emulsions.

### 3.3. A Multilayer Device for Scalable Production

**Figure 4:**
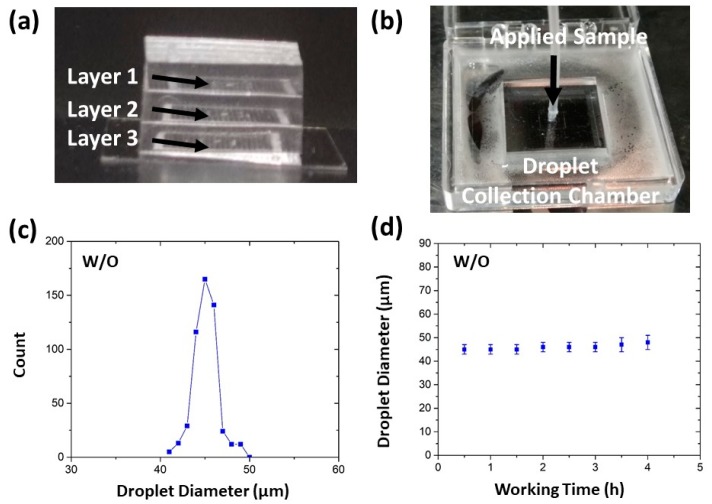
(**a**) A photograph showing a multi-layer device by stacking three layers of the HIDS generators (each layer is composed of 400 HIDS generators). (**b**) A photograph showing the operation of the multi-layer device in a collection container using only one pressure source for the dispersed phase. (**c**) The size distribution of W/O droplets of a multilayer device. (**d**) The droplet diameter remains unchanged over a duration of 4 h operation.

Our device is facile for integration for its simple layout and easy fabrication process. To further scale up the numbers of generators, we tried to integrate such devices layer-by-layer in a 3D architecture. Figure 4a,b shows the side-view and top-view photographs of the multi-layer device by stacking three layers of the HIDS generators. Video S1 (see Supplementary Materials) demonstrates the operation process of this device and uniform droplet formation from each layer, observed under a microscope. In the demo, we used Milli-Q water as the dispersed phase and HFE7500 with 1.5% (w/w) fluorosurfactant as the continuous phase. The flow rate applied at the dispersed phase was set to 1 mL/h and the continuous phase was static. The device is facile for operation as it only needs one pressure applied for the sample inlet. As the sample was pushed into the device, the sample flowed into different layers evenly and smoothly, reaching the individual droplet generator. By the self-emulsification in the oil phase, the droplets were finally collected in the holding oil chamber. As shown in Figure 4c, the formed droplets maintained 45 μm in diameter with a CV of 3%, consistent within the results from single-layer devices. Figure 4d shows the device working continuously for 4 h, and the droplet size remained unchanged. If the sample is supplied continuously and the collection chamber has sufficient room, the device can operate steadily for longer duration. 

In this operating condition, the droplet generation frequency of the multilayer device was 25 kHz for 1200 parallelized generators. Although the generate frequency is still lower compared to the integrated flow-focusing device demonstrated by Yadavali et al. using a wafer level incorporation of 10,260 microfluidic droplet generators to achieve a frequency of >1 trillion droplets per hour [23], our droplet generation rate is scalable with the number of droplet generators that can be integrated in the device. We believe such a 3D architecture has the potential to further maximize the production rate for droplet manufacturing to be applicable for industrial applications. In comparison with other high-throughput droplet generators, such as flow-focusing devices [21,22,23] and self-emulsification devices [29,30], our multiplayer device possesses many inherent merits: (1) 3D scalability in a compact size. The integrated devices of multiple layers present in previous work [21,22,23] resulted in the parallelization of droplet generators in the horizontal direction while keeping multiple flow network and fluid delivery ports in the vertical direction. Our multilayer device has extended the parallelization of droplet generators in a two-dimensional (2D) footprint to a three-dimensional (3D) architecture, making a truly scalable device in a compact size. Therefore, we can achieve a high density of droplet makers (~1200 generators in a device of 1.5 cm × 1.5 cm × 0.3 cm), which is about 5 times more than that of Yadavali et al. [23] and 3 times more than that of Nisisako et al. [21]. (2) Simple design and fabrication. Our design requires only one-step photolithography. The integration of multiple layers into a device is straightforward as it does not need precise alignment. By contrast, the parallelization of flow-focusing devices requires sophisticated design and fabrication processes such as complicated fluid network in multilayers [22], multiple inlet and outlet ports [21,22,23], and specific holder needed for precise alignment and integration [21]. The step-like [24,25,29,30], gradient [26], or edge-based [27] droplet generators require multi-step photolithography and etching to realize variations in channel depth. (3) Facile operation with minimum fluid consumption. The device operation only requires minimum control of the sample inlet as the continuous phase remains static so that we can minimize the continuous phase consumption and increase the droplet packing density. However, in those flow-focusing devices, the continuous phase flow rate is on the same order of the flow rate of the dispersed phase. For generating droplets of smaller size, the continuous phase flow and consumption must be even higher [41]. (4) Robustness and stability. Our device is not susceptible to pressure or flow fluctuations and is very robust in long-term operation. We have shown that the produced droplets remained uniform in size in a 4 h operation, and the device operation duration can be prolonged with sufficient supple. 

### 3.4. Mass Production of Microsphere Synthesis

**Figure 5:**
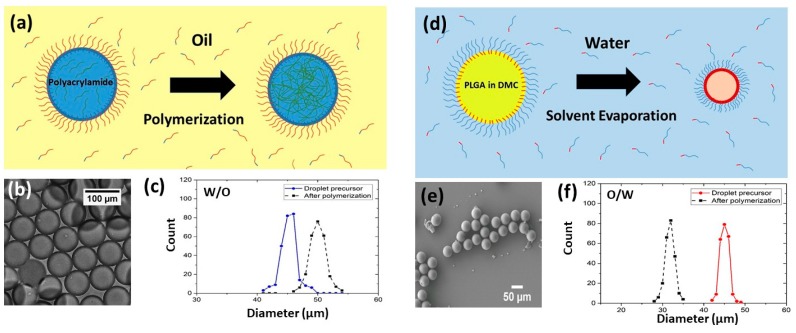
Synthesis of microspheres using both W/O and O/W emulsion templates. (**a**) Schematics of the polyacrylamide synthesis using W/O emulsion droplets. (**b**) A micrograph of the polyacrylamide hydrogel beads after polymerization. (**c**) The size distribution of the polyacrylamide hydrogel microparticles before and after polymerization. (**d**) Schematics of the Poly (l-lactide-co-glycolide) (PLGA) synthesis using O/W emulsion droplets. (**e**) A scanning electron microscope image of PLGA microparticles after the solvent evaporated. (**f**) The size distribution of the PLGA particles before and after polymerization and solvent evaporation.

To demonstrate the material compatibility and versatility of this droplet emulsification platform, we produced uniform droplets of diverse functional materials including biocompatible and biodegradable polymers and hydrogels for large-volume fabrication of solid microspheres. First, we used the W/O emulsion template for microparticles composed of hydrophilic materials such as hydrogels (Figure 5a). Such aqueous-based droplets can provide biocompatible and hydrophilic microcarriers that can be used for encapsulating bioactive agents [50], incorporating water-soluble ingredients [51], cell culture [52], and barcode-embedded beads for single-cell sequencing [37,38]. In this work, we synthesized the polyacrylamide hydrogel microspheres. Firstly, the device was immersed in HFE7500 oil with 0.4% (v/v) TEMED and 5% (w/w) fluorosurfactant. Then the acrylamide mixture was applied in the sample inlet of the device for droplet emulsification. Afterwards, the hydrogel droplets collected from the oil chamber (Figure 5b) were incubated at 65 °C for 12 h to allow the polymerization of beads. After polymerization, the size of the polyacrylamide hydrogel beads increased by 22% due to the hydrophilic properties causing it to swell in the water. Figure 5c shows the size distribution of the hydrogel beads before and after polymerization. The droplets produced by a multilayer device remained spherical in shape and uniform in size (CV ~2.7%). 

Next, we prepared microparticles composed of hydrophobic materials using O/W emulsion templates (Figure 5d). The polymer microparticles can be controlled in composition and size to cater for a wide range of applications. For example, PVA has been used for pharmaceutical applications [53]. Polyacrylic acid (PAA) is a biodegradable water-soluble polymer used for water treatment [54]. PLGA is one of the most frequently used biomaterials. Because of its biocompatibility and biodegradable characteristics, it is widely applied for drug delivery and therapeutic encapsulation [55]. For theranostic purposes, the microparticles need to be precisely controlled in size and monodispersity. In this work, we synthesized PLGA microparticles via O/W emulsification combined with solvent evaporation [56]. PLGA was dissolved in DMC. DMC is a volatile solvent that does not swell PDMS and is immiscible with water. After the droplets were generated, DMC solvent evaporated rapidly, leaving behind concentrated PLGA. It crosslinked spontaneously to polymerize into particles and thus the polymer particles shrunk in size after polymerization and solvent evaporation. Figure 5e is an SEM image showing the PLGA microspheres prepared from 5% PLGA in DMC. Although the sizes of the polymer microspheres decreased by 25%, they maintained good uniformity and spherical shape (Figure 5e). PDMS used in this study offers cheap and easy fabrication but largely limits the choice of materials that can be used in the device as some organic solvents or chemicals may swell the PDMS and damage the device [57].

## 4. Conclusions

In summary, we present a facile multilayer integration of microfluidic droplet generators to mass produce highly uniform microdroplets and microspheres. Spontaneous droplet generation is induced in a high aspect ratio rectangular channel by Plateau–Rayleigh instability. Based on our systematic studies of the effect of the HIDS geometry, flow rate and viscosity of the fluids, we concluded that under a critical capillary number, the droplet generation was very stable, and the droplet size was primarily determined by the channel width of the generator, which made the HIDS structure of great advantage for massive parallelization. By directly stacking layers of paralleled HIDS structures in a simple 3D architecture, we can easily scale up the droplet production rate using multilayers without compromising the droplet monodispersity. As proof of concept demos, using a multilayer PDMS device, we synthesized the polyacrylamide hydrogel and PLGA polymer microspheres using both W/O and O/W emulsion templates. We have shown the product rate can be up to 25 kHz for 4 h. The device can also easily be transferred to other substrate materials, such as glass, silicon or other polymers that are not susceptible or compatible to solvents and/or carrying fluids. With the merits of ultrahigh throughput and operation robustness, simple 3D integration promises the advancement of microfluidic technologies to meet the demand of the industrial manufacturing scale.

## Figures and Tables

**Table 1:** Dynamic viscosity of the glycerol fraction in Milli-Q water.

Glycerol Fraction in Water (v/v)	0%	5%	10%	15%	20%	25%	30%	35%	40%	45%	50%
Dynamic viscosity [cP]	1.005	1.034	1.383	1.444	1.985	2.094	2.569	3.199	4.046	5.213	6.856

## Supplementary Materials

The following are available online at https://www.mdpi.com/2072-666X/10/9/592/s1, Video S1: Design and operation of a multilayer droplet generator.
